# On a *Cercopithifilaria *sp. transmitted by *Rhipicephalus sanguineus*: a neglected, but widespread filarioid of dogs

**DOI:** 10.1186/1756-3305-5-1

**Published:** 2012-01-03

**Authors:** Domenico Otranto, Emanuele Brianti, Maria Stefania Latrofa, Giada Annoscia, Stefania Weigl, Riccardo Paolo Lia, Gabriella Gaglio, Ettore Napoli, Salvatore Giannetto, Elias Papadopoulos, Guadalupe Mirò, Filipe Dantas-Torres, Odile Bain

**Affiliations:** 1 Dipartimento di Sanità Pubblica e Zootecnia, Università degli Studi di Bari, Valenzano, BA, Italy; 2 Dipartimento di Sanità Pubblica Veterinaria, Facoltà di Medicina Veterinaria, Università degli Studi di Messina (Messina), Italy; 3 Department of Infectious and Parasitic Diseases and Pathology, Faculty of School of Veterinary Medicine, Aristotle, University of Thessaloniki, Thessaloniki 54 124, Greece; 4 Departamento de Sanidad Animal, Facultad de Veterinaria, Universidad Complutense de Madrid, Spain; 5 Département Systématique et Evolution, UMR 7205 CNRS, Muséum National d'Histoire Naturelle, Paris, France

**Keywords:** canine filarioids, *Cercopithifilaria*, *Cercopithifilaria bainae*, *Cercopithifilaria grassii*, *Rhipicephalus sanguineus*, vector, epidemiology, Europe

## Abstract

**Background:**

This study was aimed at investigating the distribution of a *Cercopithifilaria *sp. *sensu *Otranto et al., 2011 with dermal microfilariae recently identified in a dog from Sicily (Italy). A large epidemiological survey was conducted by examining skin samples (n = 917) and ticks (n = 890) collected from dogs at different time points in Italy, central Spain and eastern Greece.

**Results:**

The overall prevalence of *Cercopithifilaria *sp. in the sampled animal populations was 13.9% and 10.5% by microscopy of skin sediments and by PCR on skin samples, respectively. Up to 21.6% and 45.5% of dogs in Spain were positive by microscopical examination and by PCR. Cumulative incidence rates ranging from 7.7% to 13.9% were estimated in dogs from two sites in Italy. A low level of agreement between the two diagnostic tests (microscopical examination and PCR) was recorded in sites where samples were processed in parallel. Infestation rate as determined by tick dissection (from 5.2% to 16.7%) was higher than that detected by PCR (from 0% to 3.9%); tick infestation was significantly associated with *Cercopithifilaria *sp. infestation in dogs from two out of four sites. Developing larvae found in ticks were morphometrically studied and as many as 1469 larvae were found in a single tick.

**Conclusions:**

Our data suggest that, in addition to the most common species of filarioids known to infest dogs (i.e., *Dirofilaria immitis, Dirofilaria repens *and *Acanthocheilonema reconditum*), *Cercopithifilaria *sp. with dermal microfilariae should be considered due to its widespread distribution in southern Europe and high frequency in tick-exposed dogs.

## Background

Among veterinarians and parasitologists, canine filariae with haematic microfilariae (e.g., *Dirofilaria immitis, Dirofilaria repens, Acanthocheilonema reconditum *and *Acanthocheilonema dracunculoides*) are known better than those with only dermal microfilariae (e.g., *Onchocerca lupi *and *Cercopithifilaria *spp.). This is probably due to the fact that dermal filariae cause limited or no clinical alterations (with the exception of *O. lupi*) and that skin samples are difficult to collect since this procedure is invasive and thus not easily accepted by pet owners. Additionally, blood microfilariae are easily visible in blood smears during routine examination of dogs. However, cases of *O. lupi *causing acute or chronic ocular disease in dogs (i.e., conjunctivitis, photophobia, lacrimation, ocular discharge, exophthalmia) [1] have been reported in the United States [2-4], and Europe [5-8]. Likewise for many other filarioids [9], *O. lupi *has been recently been implicated as an agent of ocular zoonosis [10].

Filarioids belonging to the genus *Cercopithifilaria *Eberhard, 1980, which are transmitted by hard ticks (Ixodidae), parasitize a range of host species, including dogs [11]. Recently, dermal microfilariae of the genus *Cercopithifilaria *found in a dog from Sicily (southern Italy), *Cercopithifilaria *sp. *sensu *Otranto *et al*., 2011 (hereinafter reported as *Cercopithifilaria *sp.), were characterised morphologically and differentiated from other microfilariae commonly found in dogs [12]. The genetic identity of these microfilariae was also assessed by molecular amplification, sequencing and by a comparative phylogenetic analysis of multiple ribosomal ITS-2 and mitochondrial (*cox*1 and 12S) target genes [12]. Interestingly, the microfilariae examined in Sicily were short (mean length, 185 μm), and thus distinct from *Cercopithifilaria grassii*, a parasite described in a dog from central Italy, more than one century ago as *Filaria grassii *[13], which had exceptionally long microfilariae (660 μm). The microfilariae of *Cercopithifilaria *sp. had a similar size to those of a species described in Brazilian dogs, *Cercopithifilaria bainae *Almeida & Viente, 1984 [14]. However, since neither adult nematodes from the Sicilian case were available nor the holotype of the Brazilian species was restudied for comparison, a conclusive specific assessment of *Cercopithifilaria *sp. [12] is lacking. Nevertheless, the competence of the brown dog tick, *Rhipicephalus sanguineus*, as an intermediate host of this filarial species has been experimentally investigated and results obtained showed that these microfilariae develop to the third-stage infective larvae (L3) in nymphs [15]. Additionally, a PCR protocol for the detection of *Cercopithifilaria *sp. in dog skin samples and ticks was assessed and proposed as a tool for further epidemiological studies [12].

In past years, single reports of filarioids identified as *C. grassii *were described in ticks from Switzerland [16] and northern Italy [17] but no data on the distribution of *Cercopithifilaria *spp. in dogs and in its tick vectors are so far available in the literature. This lack of knowledge impairs the understanding of the epidemiology of *Cercopithifilaria *spp. infecting dog populations and the study of their pathogenic role to dogs, at a local (dermic) or systemic level. Thus, one year after the retrieval of the first case of *Cercopithifilaria *sp. infestation in a dog from Sicily [12], this current study was carried out to investigate the occurrence of this filarioid in selected populations of dogs exposed to *R. sanguineus *from three countries of the Mediterranean area (i.e., Italy, Spain and Greece). Skin samples from dogs enrolled in previous studies at different time points or specifically sampled from animals together with their ticks were tested microscopically and/or molecularly in order to give the first comprehensive account of the occurrence of this *Cercopithifilaria *sp. in dogs living in countries in the Mediterranean area.

## Materials and methods

### Study areas and sampling times

**Figure 1:**
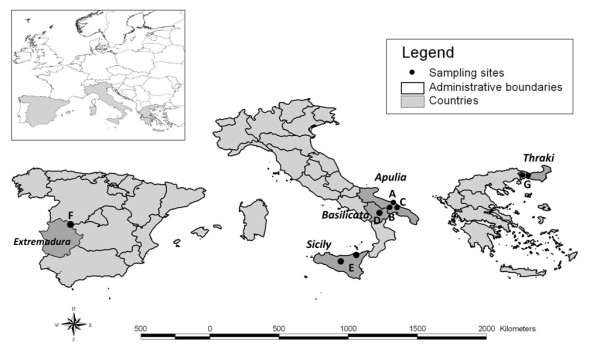
**Sampling localities**. Localities from which samples were collected in Spain, Italy and Greece.

A total of 917 skin samples were collected at different time points from dogs living in regions of southern Italy (n = 843), central Spain n = 51 (site F) and eastern Greece n = 23 (site G). Animals from Italy come from Apulia (municipalities of Bari n = 280 (site A), Ginosa n = 320 (site B) and Putignano n = 80 (site C)), Basilicata (Parco Regionale di Gallipoli Cognato - Piccole Dolomiti Lucane n = 50 (site D)) and Sicily (municipalities of Palermo and Messina n = 113 (site E)) (Figure 1).

Skin samples from Apulia (680) were collected under the context of previous studies aiming to perform a molecular diagnosis of *Leishmania infantum *[18,19]. In particular, 600 skin samples were sampled from dogs on March 2005 from site A (280) and B (320), while the remaining 80 animals were sampled on October 2009 from site C. In addition, in order to evaluate the incidence rate of *Cercopithifilaria *sp. infestation in the study populations, skin samples of some dogs were sampled again at 7 and 10 months intervals in sites B and C, respectively.

Dogs from other sites were sampled between February 2011 (site E) and July 2011 (site D), specifically for this study. All dogs sampled were naturally exposed to *R. sanguineus *infestation throughout the year [20-22] and mainly during spring and summer, which are the seasons with the higher number of ticks infesting dogs in temperate regions [23]. All animals sampled were not treated with endo- or ecto-parasiticide. During the collection of skin samples, dogs were also checked for tick presence and a total 890 *R. sanguineus *ticks, almost all adults but also some nymphs, were individually collected from animals sampled on site C (n = 257), site D (n = 77), site E (n = 272), site F (n = 66) and site G (n = 218).

The study design and the experimental procedures for skin samples collected from previous studies (i.e., sites A, B and C) were approved by the Animal Ethics Committee of the University of Bari (Italy) and authorized by the Italian Ministry of Health (authorization number 72/2009C, n°. 69062; 11/28/2008) [18,19].

### Sampling and laboratory procedures

Due to the different provenance and timing of sampling, the procedures varied slightly as detailed in the following. Skin samples were collected using a disposable scalpel from the right shoulder or inter-scapular region (about 0.5 × 0.5 × 0.6 cm) and stored directly at -20°C (sites A, B, and C) or soaked in saline solution for 10 min at 37°C (sites D, E, F, and G) and thereafter removed and stored at -20°C for the molecular analysis. When skin samples were soaked in saline solution, the sediment was observed under light microscopy (two fields of 10 × 10 mm coverslip each) after adding a drop of methylene blue (1%) whereas samples from Sites A, B, and C were tested by PCR only. Dermal microfilariae were morphologically identified [12]. Briefly, microfilariae have a rounded head and a short dorso-ventrally flattened body (mean length of 186.7 ± 3.9 μm, width 8.5-11 μm and 3-3.5 wide in lateral view). No sheath is present and the body cuticle is thick with conspicuous transverse striations. Approximately 4-5 angular nuclei are detectable in dorso-ventral view and one rounded nucleus in lateral view on a transverse line.

Ticks collected were placed on a microscope slide and individually processed in a few drops of sterile saline solution under a stereomicroscope. Once ticks have been dissected, a drop of methylene blue (1%) was added to visualize the larval nematode and a coverslip was placed on the preparation. This preparation was microscopically observed at 10 × and 40 × magnification. Larvae recovered in ticks were morphologically identified [15]; furthermore, developing forms found in ticks collected in sites D and E were also counted and staged. Following the microscopical observation and irrespective of the positivity, the remaining part of dissected ticks was stored in individual vials containing 70% ethanol for further molecular analysis (see below). Ticks collected from dogs in site C were molecularly processed only. Microfilariae found in dermal samples and developing forms found in the dissected ticks were photographed with a digital camera (Zeiss Axiocam MRc, Carl Zeiss AG, Germany) mounted on the microscope (Zeiss Axioscop 2 plus, Carl Zeiss AG, Germany) and measurements (in micrometres) were taken with the AxioVision rel. 4.8 software (Carl Zeiss AG, Germany).

### Molecular examination

Genomic DNA was extracted from skin samples using a commercial kit (DNeasy Blood & Tissue Kit, Qiagen, GmbH, Hilden, Germany) in accordance with the manufacturer's instructions, whereas from *R. sanguineus *adult ticks as described elsewhere [24]. Skin samples and ticks were individually tested. All samples were molecularly processed for specific amplification of a partial *cox*1 (~304 bp) gene fragment targeting *Cercopithifilaria *sp., using specific primers CbCox1F/NTR following reaction procedures and amplification protocol described in the literature [12]. DNA from *R. sanguineus*, blood and skin samples from laboratory-reared beagles [25] were used as negative controls, along with a no DNA sample, which were included in each reaction to test the specificity of the reaction and to assess the presence of contaminants. About 20% of amplicons were purified using Ultrafree-DA columns (Amicon, Millipore; Bedford, USA) and sequenced directly using the Taq DyeDeoxyTerminator Cycle Sequencing Kit (v.2, Applied Biosystems) in an automated sequencer (ABI-PRISM 377). Sequences were aligned using the ClustalW program [26] and compared among them and with those available in GenBank™ dataset by BLAST analysis.

### Data analysis

Differences in the frequencies of *Cercopithifilaria *infestation both in dogs or ticks in the same study site were evaluated for statistical significance by using chi-square test (with Yates' correction) or, when appropriate, by Fisher's exact test. Cumulative incidence for *Cercopithifilaria *sp. in animals from sites B and C was calculated as the proportion of new cases (molecularly detected) that occur in the two study populations over the period of months at risk [27]. Agreement between the two diagnostic tests was evaluated using Kappa (K) statistics and the proportion of agreement beyond chance expressed as values of K in a scale ranging from 0 (no agreement) to 1 (perfect agreement). The association between *Cercopithifilaria *infestation in dogs and in ticks was expressed by the odds ratio (OR) and the hypothesis tested for statistical significance by Fisher's exact test. Critical significance level (α) was set at 5% (0.05) and all tests were performed two-sided. Statistical analyses was carried out using the statistical packages GraphPad InStat v. 3.05 (GraphPad Software, Inc.) and WinEpiscope 2.0.

## Results

A total of 237 skin sediments and 877 skin samples were examined for the presence of *Cercopithifilaria *sp. by microscopic and molecular analysis, respectively. Based on the diagnostic test employed, the overall prevalence of *Cercopithifilaria *sp. in the sampled animal populations ranged from 13.9% to 10.5% by using microscopy and PCR, respectively. The higher prevalence rate of infested animals was recorded in Spain either by microscopical examination of skin sediments (21.6%) or by molecular detection on skin samples (45.5%) whereas the lower positivity rate was in Greece (4.3%). In Italy, according to the sites and to the diagnostic tests employed, the prevalence of *Cercopithifilaria *sp. infestation in dogs varied from 5.3% (site B) up to 19.5% in site E (Table 1).

**Table 1:** Number and percentage (in brackets) of dogs and ticks positive for *Cercopithifilaria *sp. divided according to the geographical site of collection and diagnostic method used (i.e., microscopical examination of skin sediment or molecular analysis of skin samples)

		Dogs			Ticks		
		
Country	Locality/Region	Microscopical examination	Molecular analysis	*P*	Microscopical examination	Molecular analysis	*P*
		
		pos/tot (%)	pos/tot (%)		pos/tot (%)	pos/tot (%)	
Italy							
	Bari (A)	-	38/280 (13.6%)	-	-	-	-
	Ginosa (B)	-	17/320 (5.3%)	-	-	-	-
	Putignano (C)	-	10/80 (12.5%)	-	-	10/257 (3.9%)	-
	Basilicata (D)	6/50 (12%)	0/50 (0%)	0.026*	4/77 (5.2%)	2/77 (2.6%)	0.6812*
	Sicilia (E)	15/113 (13.3%)	22/113 (19.5%)	0.2808	41/272 (15.1%)	7/272 (2.6%)	< 0.0001
Spain							
	La Vera (F)	11/51 (21.6%)	5/11 (45.4%)	0.132*	11/66 (16.7%)	0/66 (0%)	0.0016
Greece							
	Xanthi (G)	1/23 (4.3%)	0/23 (0%)	1*	14/218 (6.4%)	1/218 (0.5%)	0.0016

*P *values indicate the statistical significance of differences between test frequencies as determined by Yates' corrected chi-square test or Fisher's exact test (*) when appropriate (α = 0.05).

No difference was detected in the frequency of positive samples from the same site as determined by the two diagnostic methods except in site D (*P *= 0.0266) where PCR produced no positive result. A significant difference in the frequency of infection between dog sexes was only found in site A where male dogs showed a higher rate (23.4%) of infestation than females (7.5%; *P *= 0.0003). The incidence of infestation was calculated in dogs from sites B and C. Indeed, a cumulative incidence of 7.7% (8 out 104 animals over 10 months at risk) and of 13.9% (5 out 36 animals over 7 months at risk) was estimated in sites B and C, respectively (data not shown).

A low level of agreement between the two diagnostic tests was recorded in sites where samples were tested by microscopy and PCR. The higher proportion of agreement (K = 0.241) was recorded in samples from site (F) where 2/5 of PCR-positive samples were also positive by microscopy and 1/6 of PCR-negative samples were positive by microscopical examination. In site E, six out of 22 PCR-positive samples were also positive by microscopical examination, whereas 9 out of 91 PCR-negative samples were positive by microscopical examination (K = 0.198) (Table 2). No concordance was observed between the two diagnostic tests in sites D and G, where all the samples processed by PCR were negative.

**Table 2:** Agreement between diagnostic tests: number of positive and negative samples examined in parallel by microscopical examination of skin sediment and molecular analysis of skin for *Cercopithifilaria *sp. infection in Sicily (Site E) and Spain (Site F)

	Microscopical examination	Molecular examination		Total
			
		Positive	Negative	
Sicily (E)	Positive	6	9	15
	Negative	16	82	98
	Total	22	91	113
				

	**Microscopical examination**	**Molecular examination**		**Total**
			
		**Positive**	**Negative**	

Spain (F)	Positive	2	1	3
	Negative	3	5	8
	Total	5	6	11

**Figure 2:**
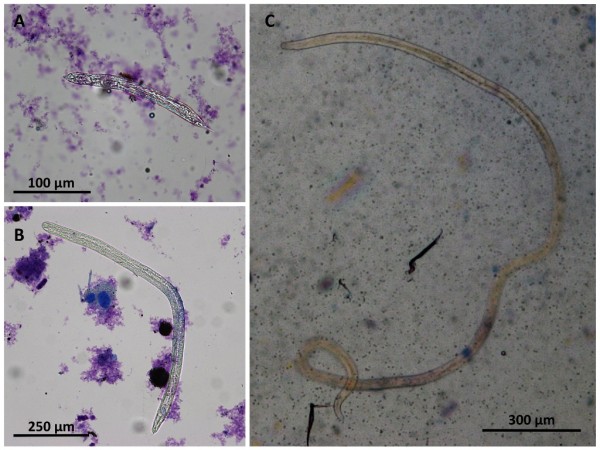
**Larval *Cercopithifilaria *sp. in dissected ticks**. Developing forms of *Cercopithifilaria *sp. found in dissected ticks: A) First stage larva; B) Second stage larva; C) Third stage infective larva. Ticks were dissected in few drops of saline solutions and larvae stained with methylene blue (1%).

Prevalence of infestation in ticks collected ranged from 5.2 to 16.7% and from 0% to 3.9% by using dissection and PCR, respectively (Table 1). In all cases, the positivity rate determined by tick dissection was higher than that by molecular examination. With the exception of site D, prevalence of infested ticks determined by microscopy was correlated with those obtained by the microscopical examination of skin sediments of dogs from the same site. In addition, tick infestation was significantly associated with *Cercopithifilaria *sp. infestation in dogs from sites D (OR = 11.9, *P *= 0.0184) and E (OR = 16.2, *P *< 0.0001) (Table 3). Infected ticks were collected from both positive (38/90, 42.2%) and negative (52/90, 57.8%) dogs. All developing forms found in ticks were consistent with those of *Cercopithifilaria *sp. at different stages described (Figure 2). Larval forms detected in ticks were counted and staged in 45 positive ticks (i.e., 4 of site D and 41 of site E) with the most frequent stage being found were microfilariae (mfs) (84.4%) (See additional file 1: Microfilaria of *Cercopithifilaria *sp. in ticks), followed by developing L1 (11.1%), L2 (6.7%) and L3 (6.7%) (Table 4). As many as 1469 larvae (i.e., 1453 mfs, 11 L1 and 5 L2) of *Cercopithifilaria *sp. were found in a single tick (See additional file 2: Second stage larvae of *Cercopithifilaria *sp. in ticks). Interestingly, 16 (11 males and 5 females) out of the 38 ticks harbouring the mfs stage of *Cercopithifilaria *sp. were collected from dogs negative by microscopical examination of skin sediment.

**Table 3:** Association between *Cercopithifilaria *sp. infestation in dogs (positive/negative) and the presence or absence of tick infestation on the same animals from the different sites examined

Locality/region	*N*	Tick-infested dogs		Tick-free dogs		Odds ratio (95% CI)	*P*
				
		positive	negative	positive	negative		
Putignano (C)	80	6	24	4	46	2.9 (0.7-11.2)	0.1640
Basilicata (D)	50	5	13	1	31	11.9 (1.3-112.3)	0.0184
Sicilia (E)	113	13	28	2	70	16.2 (3.4-76.7)	< 0.0001
Madrigal de la Vera (F)	51	9	32	2	8	1.1 (0.2-6.3)	1.000
Xanthi (G)	23	1	8	0	14	5.1 (0.2-14.4)	0.3913

**Table 4:** Number and percentage of infected ticks, mean intensity, range of infestation and measurements of larval *Cercopithifilaria *sp. according to their developmental stage

Developing forms	% Tick infected (pos/tot)	Mean intensity (± S.D.)	Range	Measurements (microns)	
				
				Length (± S.D.)	Width (± S.D.)
mfs	84.4 (38/45)	49.6 (± 237.5)	1-1453	192.2 (± 9.9)	5.6 (± 1)
L1	11.1 (5/45)	6.8 (± 6.2)	1-15	229.2 (± 16.6)	19.2 (± 4.4)
L2	6.7 (3/45)	12.3 (± 13.7)	2-27	724 (± 13.6)	27.1 (± 1)
L3	6.7 (3/45)	8.3 (± 12.7)	1-23	1706 (± 61)	26.5 (± 1.5)

Tick samples constituted a total of 45 positive ticks collected in Basilicata (Site D) and Sicily (Site E). Measurements of length and width of developing larvae in ticks are expressed as mean calculated on 10 microfilariae (mfs), 7 L1, 7 L2 and 5 L3.

## Discussion

This study represents the first survey to investigate the occurrence of this recently discovered *Cercopithifilaria *sp. in dogs from selected geographical areas of the Mediterranean basin and in ticks collected from the same animals. Indeed, as a main conclusion for the first report of the *Cercopithifilaria *sp. with dermal microfilariae in a dog from Sicily [12], it was not clear whether this was an occasional finding or a widespread, though neglected, canine infestation. Nonetheless, considering the widespread distribution of *R. sanguineus *[28], which is regarded as the putative vector of this filarioid [15], it was suggested that this species could be a common parasite of dogs in temperate areas. Accordingly, this study indicates that the *Cercopithifilaria *sp. here investigated is widespread in dogs from the Mediterranean countries surveyed with an overall prevalence ranging from 10.5% to 13.9%. These prevalence rates are higher than those detected in large epidemiological surveys for other filarioids with the exception of *D. repens *in dogs living in hyper-endemic areas of southern Italy, where a prevalence of microfilaraemic dogs of up to 30.8% were recorded [29]. In particular, the highest prevalence rate of infested animals was recorded in Spain (from 21.6% up to 45.5% by microscopical examination or molecular detection of skin samples) whereas the prevalence of infestation in Italy (up to 19.5%) was close to the mean values. The significant difference in the frequency of infection in male dogs (23.4%) compared to females (7.5%) found in site A might reflect the male-biased infestation by *R. sanguineus *in some areas, as demonstrated in a confined dog population in southern Italy [21].

The cumulative incidences of 7.7% over ten months and 13.9% over seven months were recorded in sites B and C, respectively. In both sites incidence was calculated after the spring and summer seasons, which are the seasons with the highest number of *R. sanguineus *ticks infesting animals in the Mediterranean region and, specifically, in the study sites [21]. Interestingly, even if dogs from site C were exposed to ticks for a shorter period than dogs from site B, a high incidence rate was detected in site C. This finding could be explained by the higher rate of *Cercopithifilaria *sp. infestation in dogs from site C (12.5%) than that from site B (5.3%). However, both cumulative incidence values recorded in this study were very high if compared to the force of infection, which expresses the probability of a susceptible dog becoming infected in one year, calculated across European countries for *D. immitis *(up to 8.4%) and *D. repens *(up to 3.3%), respectively [30].

A low level of agreement between the two diagnostic methods (i.e., microscopical examination versus PCR) was recorded in this study, considering either skin samples or ticks. In addition, in all ticks and skin samples from sites D and G that were tested in parallel, the rate of infestation was higher when determined by microscopical examination than by PCR. This discordance might be due to the fact that all microfilariae present in the skin samples had deposited on the bottom of the eppendorf tubes, being easily detected by microscopical examination of the sediment. Consequently, the skin piece used for DNA extraction and subsequent PCR testing had less or no microfilariae. Similarly, all developing larvae detected in ticks were isolated, singularly observed, photographed and thus the remaining tissues of the dissected tick that was used for molecular testing had less or no microfilariae as well.

The occurrence of developing second and third-stage larvae of *Cercopithifilaria *sp. in *R. sanguineus *after feeding on an infested dog, their morphological similarity with those of *Cercopithifilaria *genus [13,16,17] and molecular homology with the dermal microfilariae of *Cercopithifilaria *sp. described from the same dog [12] suggested that this tick species may represent a competent intermediate host [15]. The positivity for *Cercopithifilaria *sp. recorded in ticks collected from sampled dogs (from 5.2 to 16.7% and from 0.5 to 3.9% by using dissection and PCR), provides circumstantial evidence indicating that *R. sanguineus *is the vector for this filarioid under natural conditions. Analogously, in sites where the highest number of ticks was collected on dogs (i.e., site D and E) tick infestation was significantly associated with *Cercopithifilaria *sp. infestation (Table 3). These data along with the evidence of the presence of mfs and developing second- and third-stage larvae in ticks, further indicate that *R. sanguineus *may act as vector of this *Cercopithifilaria *sp. in nature.

Indeed, all developing forms found in ticks were similar to the different stages described elsewhere for this *Cercopithifilaria *sp. [15]. Interestingly, the extraordinary finding of up to 1469 developing forms of *Cercopithifilaria *sp. in one tick (i.e., 1453 mfs, 11 L1 and 5 L2) suggested that *Cercopithifilaria *sp. infestation is well tolerated by *R. sanguineus *and that the tick viability is not impaired by the nematode larvae. This could represent the foundation for further investigations on the *Cercopithifilaria *sp.-vector relationship and to explain the broad distribution of this nematode among tick-infested dogs. The finding that both male and female ticks positive for *Cercopithifilaria *sp. were collected from both positive (38/90, 42.2%) and negative (52/90, 57.8%) dogs might indicate that both sexes act as vectors of this nematode. In particular, the multiple-feeding behaviour of male ticks on the same dog or on co-housed dogs and the long periods of time they spend on the host [31] increase their potential as vectors of pathogens (e.g., *Ehrlichia canis*, ref. 32), including *Cercopithifilaria *sp.

Our data also suggest that the xenodiagnosis (i.e., detection of larvae in their vectors) might be useful for detecting skin-dwelling microfilariae and should even be preferred to the direct observation of skin samples, if the aim of study is to detect the occurrence of this parasite in a given animal population. Whereas, the examination of skin samples should be preferred when the diagnosis is requested at the individual level. In addition, in *post mortem *studies, soaking carcasses and skin strips in warm saline (soon after the animal death) followed by a series of decantation, is efficacious in retrieving adult and larval worms [33]. However, this procedure is not easy to perform, it is time consuming, and not acceptable for the majority of owners.

## Conclusions

Finally, the scientific evidence presented here suggests that, in addition to the most common species of filarioids known to infest dogs (i.e., *D. immitis, D. repens *and *A. reconditum*), *Cercopithifilaria *sp. with dermal microfilariae should also be considered. Although microfilariae are useful to morphologically differentiate onchocercid species, no definitive conclusion on the identity of this species can be made in the absence of other nematode stages, particularly adults. Nonetheless, the microfilariae here retrieved are morphologically close to those of *C. bainae *(i.e., 185.18 μm and 6.59 μm in length and width, respectively [34]). Future studies should prioritize investigations on the specific identity of this parasite, its pathogenicity at a local (dermal) and/or systemic level and the potential for antigenic cross-reactivity with other onchocercids of dogs. Undoubtedly, the genetic make-up of *Cercopithifilaria *spp. and their genetic affiliation with the vector and the host might provide interesting information on the role *R. sanguineus *in disseminating *Cercopithifilaria *onchocercids within animal populations.

## Competing interests

The authors declare that they have no competing interests.

## Authors' contributions

DO, conceived the research, collected samples, contributed with data analysis and interpretation and wrote the first draft of the manuscript. EB, FDT, collected samples, contributed with data analysis, interpretation and revision of the manuscript. OB, contributed with data analysis, interpretation and revision of the manuscript. MSL, SW and GA run the molecular assays. GM and EP collected samples, contributed with data analysis and interpretation and revision of the manuscript. RPL, GG and EN collected samples and dissected ticks. All authors read and approved the final version of the manuscript.

## Supplementary Material

Additional file 1 **Microfilaria of *Cercopithifilaria *sp. in ticks**. Microfilaria of *Cercopithifilaria *sp. found in a dissected tick. Click here for file

Additional file 2 **Second stage larvae of *Cercopithifilaria *sp. in ticks**. Second stage larvae of *Cercopithifilaria *sp. found at tick dissection. Note the presence in the same field of numerous microfilariae of the same species. Click here for file
